# Specialization directs habitat selection responses to a top predator in semiaquatic but not aquatic taxa

**DOI:** 10.1038/s41598-021-98632-2

**Published:** 2021-09-23

**Authors:** Hana Šigutová, Filip Harabiš, Martin Šigut, Jiří Vojar, Lukáš Choleva, Aleš Dolný

**Affiliations:** 1 Department of Biology and Ecology, Faculty of Science, University of Ostrava, 71000 Ostrava, Czech Republic; 2 Department of Ecology, Faculty of Environmental Sciences, Czech University of Life Sciences Prague, 16521 Prague, Czech Republic; 3 Laboratory of Fish Genetics, Institute of Animal Physiology and Genetics, Czech Academy of Sciences, 277 21 Liběchov, Czech Republic

**Keywords:** Behavioural ecology, Biodiversity, Freshwater ecology

## Abstract

Habitat selectivity has become an increasingly acknowledged mechanism shaping the structure of freshwater communities; however, most studies have focused on the effect of predators and competitors, neglecting habitat complexity and specialization. In this study, we examined the habitat selection of semiaquatic (amphibians: Bufonidae; odonates: Libellulidae) and aquatic organisms (true bugs: Notonectidae; diving beetles: Dytiscidae). From each family, we selected one habitat generalist species able to coexist with fish (*Bufo bufo*, *Sympetrum sanguineum*, *Notonecta glauca*, *Dytiscus marginalis*) and one species specialized in fishless habitats (*Bufotes viridis*, *Sympetrum danae*, *Notonecta obliqua*, *Acilius sulcatus*). In a mesocosm experiment, we quantified habitat selection decisions in response to the non-consumptive presence of fish (*Carassius auratus*) and vegetation structure mimicking different successional stages of aquatic habitats (no macrophytes; submerged and floating macrophytes; submerged, floating, and littoral-emergent macrophytes). No congruence between habitat specialists and generalists was observed, but a similar response to fish and vegetation structure defined both semiaquatic and aquatic organisms. While semiaquatic generalists did not distinguish between fish and fishless pools, specialists avoided fish-occupied pools and had a preferred vegetation structure. In aquatic taxa, predator presence affected habitat selection only in combination with vegetation structure, and all species preferred fishless pools with floating and submerged macrophytes. Fish presence triggered avoidance only in the generalist bug *N. glauca*. Our results highlight the significance of habitat selectivity for structuring freshwater ecosystems and illustrate how habitat selection responses to a top predator are dictated by specialization and life history.

## Introduction

Oviposition habitat selectivity is an important mechanism for structuring populations, communities, and metacommunity assemblages of aquatic organisms^1,2^. Traditionally, community assembly was perceived to result from random dispersal followed by non-random, site-specific competition, resource-related mortality, and predation (i.e., species sorting)^1–3^. However, empirical evidence suggests that these post-colonization processes may be obviated, or at least co-determined, by habitat selection^1,4–6^, whereby species actively colonize patches providing the highest expected fitness^7,8^. Therefore, the resulting spatial redistribution of individuals among habitat patches is based on perceived rather than realized fitness, driven by habitat interactions in the species’ evolutionary past^1,9,10^. Predators are unevenly distributed within most landscapes, where they negatively affect prey fitness^11,12^. The ability to avoid predator-occupied patches during colonization and oviposition would increase offspring survival and, consequently, their fitness^7,13,14^.

The presence of predaceous fish dictates successful colonization by most freshwater taxa^15,16^. The ability and mechanisms for avoiding fish habitats, however, vary based on morphological or physiological features and general evolutionary adaptations. Oviposition site selection in response to predation risk is likely to evolve if: (1) immature individuals are subjected to high predator-induced mortality risk; (2) females can oviposit on a number of patches; (3) predator distributions among patches are random but fixed from oviposition until the progeny can leave the patch^4^. These conditions are often encountered in taxa with aquatic larval stages and highly mobile terrestrial adults, such as amphibians or semiaquatic insects, or in aquatic insects, whose winged mobile imagoes colonize new habitats^4,13^. Fish avoidance has been documented in amphibians^6,9,17,18^, beetles^1,6,13^, dipterans^6,19^, and true bugs^13^. In contrast, odonates seem be unresponsive to the presence of fish^20–22^, but experimental studies have been limited to only a few habitat generalist species.

Habitat selection is based on highly specific visual, tactile, and chemical cues, or their combination^1,2,13,23^. Amphibians, as well as aquatic and semiaquatic insects, base their visual habitat recognition on the structure of macrophyte vegetation^24–26^; moreover, these insects locate their habitats primarily by detecting horizontally polarized light reflected from the water surface, which is influenced by depth, turbidity, and transparency^27,28^. Predators may be detected directly via chemoreceptors^23,29^ or through indirect visual cues, such as vegetation structure^25,26,30^, to indicate absence of fish^14^. Vertebrate predators are typically absent from early-successional or temporary habitats lacking developed aquatic macrophyte vegetation^26,31^ or acidic habitats rich in sphagnum moss^32^ and are associated with permanent habitats with complex vegetation^4^. By relying on indirect cues, predators can be avoided without being encountered. However, a mismatch may arise between cue-based preferences and habitat quality, especially in modified habitats^33^.

The cues used for habitat selection are complex and difficult to discern^34^. Yet, most studies in freshwater ecosystems considered predators or competitors as the only cue (but see^1^). Moreover, such studies were based on field observations under uncontrolled parameters or on experiments in naturally colonized landscapes, and targeted habitat specialists for which significant selectivity was expected^23^. As a result, the habitat cues and mechanisms crucial for understanding the role of animal behavior in the formation of natural freshwater communities and integration in (meta)community models remain unknown^10,35^. Thus, more complex experiments are needed to understand the colonization patterns of freshwater communities.

In this study, we examined the colonization and oviposition behavior of highly vagile semiaquatic (amphibians: Bufonidae, odonates: Libellulidae) and aquatic organisms (true bugs: Notonectidae, diving beetles: Dytiscidae). From each family, we selected one habitat generalist species able to coexist with fish (*Bufo bufo*, *Sympetrum sanguineum*, *Notonecta glauca*, *Dytiscus marginalis*) and one specialized species that strongly preferred fishless habitats (*Bufotes viridis*, *Sympetrum danae*, *Notonecta obliqua*, *Acilius sulcatus*). We quantified their preferences in response to the non-consumptive presence of fish and vegetation structure mimicking different successional stages of aquatic habitats. We hypothesized that generalists would not show selectivity for predator-occupied and predator-free patches because they and/or their progeny either displayed constitutive anti-predator defenses (e.g., spines, unpalatability, toxicity, defensive secretions)^36–38^, or reacted morphologically and/or behaviorally to predator cues^39–41^. We had two hypotheses concerning specialists. The first one was that they and/or their progeny were highly vulnerable to predation due to the absence of defensive and compensatory mechanisms; hence, making them favor fishless habitats with the vegetation structure matching their habitat preferences. The second hypothesis was that specialists recognized their preferred habitat based on vegetation structure, but because it inherently had no fish, they would not be able to detect predators^3^. By creating a mismatch between indirect cues indicating predator presence/absence and its real presence/absence, our design allowed us to (1) quantify the effects of predator cues and vegetation structure on habitat selection, and (2) disentangle the effect of habitat specificity from the actual ability to detect predators.

## Methods

### Study species

The common toad *Bufo bufo* (Linnaeus, 1758) (Anura: Bufonidae) is a widespread generalist. Highly poisonous bufotoxins in the skin of adults and tadpoles are an effective defense against vertebrate predators^36^, allowing spawning in large permanent water bodies with fish^26^. The green toad *Bufotes viridis* (Laurenti, 1768) inhabits warm and arid lowland regions, such as steppes, riverbanks or man-made structures (e.g., quarries, agricultural land, and urban areas). The spawning sites of this specialist include shallow, often ephemeral and warm water bodies, such as flooded fields, pools, or ditches, typically without fish and vegetation^42–44^.

The dragonfly *Sympetrum sanguineum* (Müller, 1764) (Odonata: Libellulidae) is a broadly distributed habitat generalist that inhabits all types of stagnant water bodies^45^. Its larvae develop dorsal and lateral spines on the abdomen that provide protection against fish^38^. The specialist *Sympetrum danae* (Sulzer, 1776) prefers acidic waters, such as peat bogs, fens, and moors; however, it may also inhabit shallow, densely overgrown ponds and ditches, particularly if associated with sedge, rush, and sphagnum moss^45^. Similar to other odonate species specialized in fishless habitats, the abdominal spines of its larvae are reduced, making them vulnerable to fish^46^.

The generalist aquatic true bug *Notonecta glauca* Linnaeus, 1758 (Hemiptera: Notonectidae) inhabits a wide range of habitats^47,48^, preferably with vegetation and including fish^48^. The specialist *Notonecta obliqua* Thunberg, 1787 is considerably less common and prefers bogs and fens with acidic water^49,50^. Its preference for fishless habitats, as well as dark body coloration, suggests high vulnerability to fish predation^51^.

The large diving beetle *Dytiscus marginalis* Linnaeus, 1758 (Coleoptera: Dytiscidae) is found in numerous aquatic habitats but prefers relatively deep, open waters. Because of its large body (27–35 mm), short lifespan as larvae, a hard cuticle, and defensive secretions, this generalist species is resistant to fish predation^37^. The specialist *Acilius sulcatus* (Linnaeus, 1758) is a smaller dytiscid species (15–18 mm). In spite of secreting defensive vertebrate-type steroids^52^, adults are highly susceptible to fish predation^53^ and readily respond to fish chemical stimuli^54^. This species prefers larger water bodies with rich submerged vegetation^55^ but may also colonize temporary habitats or those in early successional stages to escape predation by fish^54,56^.

### Design of the mesocosm experiment

The study was conducted in 2019–2020 during the period of epigamic activity of the study species (see below). The experimental array consisted of 24 plastic tanks (110 cm diameter, 35 cm depth, 275 L) arranged in four spatial blocks of six pools each (see Supplementary Fig. S1 online). Individual blocks were represented by four experimental arenas (outdoor net cages, each of the dimensions of 12 × 6 × 3 m; steel construction covered with polyamide netting with mesh size 2 × 2 mm) located in the botanical garden of the University of Ostrava, Czech Republic (49.8274 N, 18.3259 E). Tanks within a block were arranged in two rows (three pools per row), spaced approximately 2 m apart (see Supplementary Fig. S2 online), and filled with well water. Tanks were surrounded by grass, upright branches that served as perches for dragonflies, and evenly distributed toad shelters composed of old wood and stone.

Colonists/ovipositors were sampled according to a fully randomized 2 (fish or fishless pools) × 3 (no macrophytes; only submersed and floating macrophytes; submerged, floating, and littoral macrophytes) factorial design. The six treatments (presence or absence of fish × one of three vegetation types) were randomly assigned to tanks within each block. All tanks contained plastic predator cages (40 cm diameter × 40 cm height) covered with a polyethylene screen (mesh size of 5 × 5 mm), allowing larger prey to pass through while providing visual and chemical cues indicating the presence of fish to experimental organisms, but preventing fish from consuming them. Fish were represented by three 15–20-cm-long individuals of the crucian carp *Carassius auratus* (Cyprinidae). This invasive, omnivorous predator of nymphs and adults of aquatic insects, as well as eggs and early amphibian larval stages, is typically found in stagnant water bodies in Europe^57^. Submerged and floating macrophytes were represented by *Nymphaea alba, Nuphar lutea, Elodea canadensis*, *Trapa natans*, and *Potamogeton natans,* which were distributed evenly throughout the particular pools. Littoral (emergent) macrophytes were distributed along the pool edges, and consisted of *Iris pseudacorus*, *Eleocharis palustris, Juncus* spp., and *Carex* spp. The macrophytes were collected in the field, thoroughly washed, and carefully examined to prevent uncontrolled colonization. Vegetation levels, composition, and arrangement remained constant in fish and fishless pools.

Prior to starting the experiment (March, 2019), each pool was inoculated with detritus and organisms collected from aquatic habitats near the experimental site to provide prey for diving beetles and bugs, according to Briers and Warren^58^. A second inoculation was performed in July 2019, prior to starting the experiment with true bugs. Prey for adult dragonflies (flying insects, mainly Diptera and Lepidoptera) was captured in the adjacent meadows using a sweep net, and released evenly into each block approximately twice a week throughout the experimental period. Toads were fed by releasing laboratory-reared crickets (2 L per block) at the beginning of the experiment. Fish were fed common pelleted fish food.

### Animal experimental setup and data sampling

Habitat selection of diving beetles was monitored from May 27 to June 30, 2019, which included the period of dispersal colonization flights of the study species^59^. Prior to sampling, 52 *D. marginalis* individuals were released into two blocks (26 per block), and 106 *A. sulcatus* in the other two blocks (53 per block) to avoid *D. marginalis* preying on smaller *A. sulcatus*^60^. The beetles were randomly divided into three equally populous groups, each of which was released onto one of three shallow trays (approximately 20 × 20 cm) placed between each pair of pools within a block. The trays held only a small amount of water to promote the dispersal of beetles. Habitat selection was examined approximately every three days, for a total of 11 sampling events, by removing all macrophytes and carefully checking for beetles using hand nets (0.5 cm and 1 mm mesh). Beetles were counted, transferred to a single container, and after examining all pools within a block, they were released following the same procedure as during initial stocking to allow for de novo selection. After the fifth sampling, *D. marginalis* individuals were relocated to the blocks originally inhabited by *A. sulcatus* and vice versa*,* to ensure a balanced experimental design. All blocks were examined on the same day.

Habitat selection of true bugs was monitored from August 8 to September 9, 2019 during the period of epigamic activity and colonization flights of study species^61^ (nine sampling events). The blocks were stocked with 84 N*. glauca* individuals (21 per block) and 84 N*. obliqua* individuals (21 per block). As intrageneric predation is unlikely among similar-sized true bugs^62^, both species were kept in all four blocks simultaneously. Release and sampling were as in the case of beetles, except for the unnecessary species switch.

Habitat selection by the dragonfly *S. danae* was monitored from August 8 to 26, 2020 (nine sampling days; 32 tandem pairs, see below), and by *S. sanguineum* from August 28 to September 8 (eight sampling days; 31 tandem pairs). Two blocks were stocked with adult males (eight per block) and two blocks with adult females (eight per block) to avoid male sexual harassment impacting negatively on female fitness^30^. Habitat selection was assessed directly by observing ovipositing tandem pairs, whereby the female drops eggs directly into the water or sediment by performing abdominal dips in the air^45^. As the eggs within a clutch may be spread among several water bodies^30^, each such move is considered a habitat selection event. Observations were made around noon (between 10 and 14 h mean solar time), coinciding with the species peak epigamic activity^45^. The experiment was carried out one block at a time, with one tandem pair per observation. Each female from a “female” block was marked on the wings with a permanent marker and released into a “male” block. There, it was usually grasped almost immediately by one of the perching males, and copulation began, followed by oviposition into the pools. After oviposition, the female was returned to the female block. The mated male from the tandem pair was marked, released into the second “male” block, and replaced by an unmated male from that block to maintain constant numbers within a block and the same possible disturbance levels from other males. The same procedure was repeated until all females and males were mated.

Habitat selection by *B. bufo* was assessed from May 9 to 20, 2020 during the period of epigamic activity ^42^ and was preceded by the release of 12 individuals (six males and six females) into each block. The animals were evenly placed in their ground shelters. We were unable to obtain *B. viridis* females. However, males tend to select and occupy particular pools, and attract females through calling, usually leading to amplexus formation and oviposition^63^. Given the strong correlation between male calling and oviposition site^18^, male habitat selection was considered as determinative and was monitored from June 4 to July 5, 2020 (males called during the whole period) with 12 males per block. For each sampling event (six in *B. bufo*; 12 in *B. viridis*; sampling every 2–3 days), toads were caught by hand or by hand nets and placed in a single container. Habitat selection was considered to occur when an individual called in the immediate vicinity or from inside of the pool, or was present inside the pool without calling. After examining all pools within a block, individuals were released following the same procedure as during the initial stocking to enable de novo habitat selection. In *B. bufo*, oviposition events coincided with the habitat selection of males. In the subsequent analysis, only the habitat selection of males was considered (i.e., female choice and egg masses were not taken into account) so that the results were comparable with those obtained for *B. viridis*.

### Statistical analysis

Despite the large number of individuals used in the experiment, individual sampling events were not independent; each individual entered the experiment repeatedly and was only allowed to select among the six treatments within a block, without an opportunity to choose pools from other blocks (see Supplementary Fig. S1 online). Therefore, we used the generalized estimating equations (GEEs) for fitting marginal generalized linear models as they increase the model fit by accounting for correlations between variables^64^. The geeglm function, which has a syntax similar to glm but relies on a quasi-likelihood function instead of using full likelihood estimates, was applied to correlate datasets by fitting GEEs via the 'geese.fit' function of the 'geepack' package^65^. For all taxa, models with a Poisson distribution of errors (link = log) and exchangeable correlation structure were performed. In each model, predator presence (fish/no fish) and vegetation type (no macrophytes; submerged, floating; submerged, floating + littoral) and their interaction were always independent variables. The response variable was the number of individuals from each sampling event that selected specific pools, or the number of dips females performed during oviposition (in odonates). Identification of the sampling event (id) was used to specify individual clusters. An analysis of variance that compares models through Wald tests was used to get the most parsimonious model. The statistical significance level was set as 0.05. All analyses were performed in R version 4.0.2^66^.

### Ethics declaration

#### Approval for animal experiments

This study was carried out in compliance with the ARRIVE guidelines. Fish and amphibian handling followed the guidelines of the European Union Directive 2010/63/EU for the protection of animals used for experimental and other scientific purposes, the “Guidelines for the treatment of animals in behavioural research and teaching”. Animals were handled by LC awarded the Certificate of competency according to §17 of the Czech Republic Act No. 246/1992 coll. on the Protection of Animals against Cruelty (Registration number CZ 02361), provided by the Central Commission for Animal Welfare. The fish originated from a local fishery. The toad species collected for this study during rescue transfers were used under the permit no. 26174/ZP/2015-Br-7 issued by the Regional Office of the Hradec Králové region, following the national legislation of the country concerned (i.e., an exception according to §56 of the Czech Republic Act No. 114/1992 coll. which authorizes the manipulation of these species). Animals were housed under outdoor conditions and the same conditions followed water temperature. No mortality or stress was observed. At the end of the study, fish were released into local ponds, while amphibians were released to new replacement sites following permit no. 26174/ZP/2015-Br-7. We declare that all other manipulations with animals were performed in accordance with relevant guidelines, regulations and ethics.

## Results

**Figure 1:**
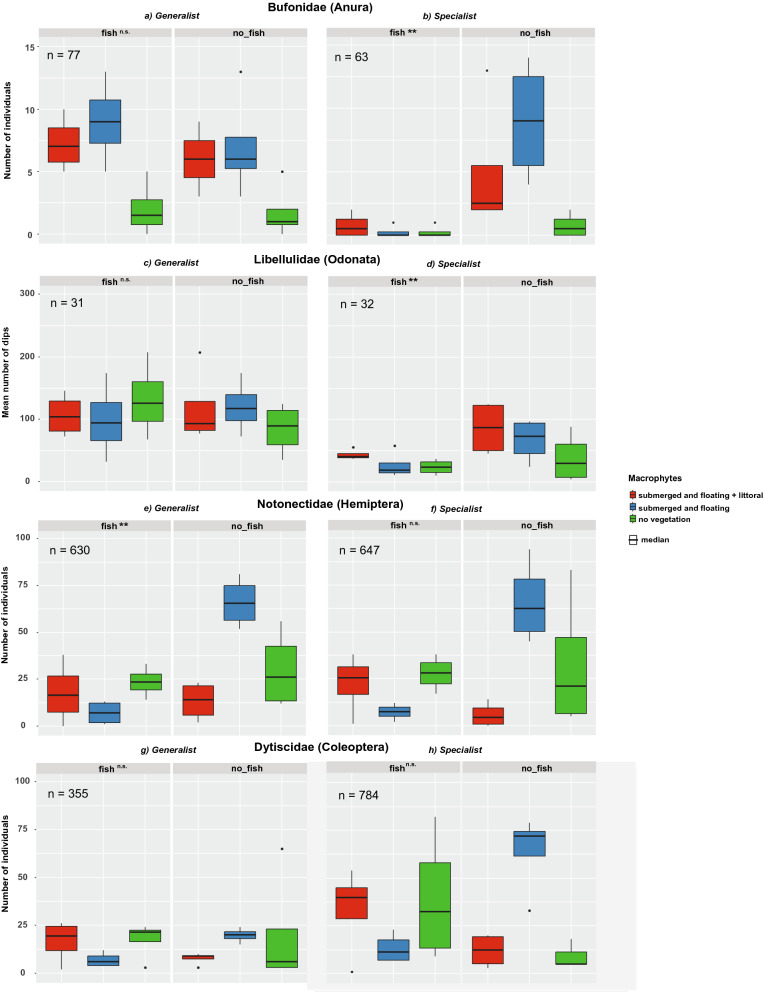
Habitat selection of semiaquatic and aquatic taxa in response to the non-consumptive presence of fish (*Carassius auratus*) and vegetation structure mimicking different successional stages of aquatic habitats. Effect of a fish (presence/absence) and vegetation structure (no macrophytes; submerged and floating macrophytes; submerged, floating, and littoral – emergent – macrophytes) on habitat selection by: (**a**, **b**) toads (Bufonidae); (**c**, **d**) odonates (Libellulidae); (**e**, **f**) true bugs (Notonectidae); and (**g**, **h**) diving beetles (Dytiscidae). (**a**) *Bufo bufo*, (**c**) *Sympetrum sanguineum*, (**e**) *Notonecta glauca*, and (**g**) *Dytiscus marginalis* represent habitat generalists, whereas (**b**) *Bufotes viridis*, (**d)** *Sympetrum danae*, (**f**) *Notonecta obliqua*, and (**h**) *Acilius sulcatus* represent more sensitive habitat specialists naturally occurring in fishless environments. In toads, true bugs, and diving beetles, *n* represents the number of individuals included in the analysis. In odonates, habitat selection was measured as the number of oviposition dips performed by a female, and *n* denotes the number of tandem pairs used in the experiment. Levels of significance between treatments with and without fish are indicated: n.s. = not significant (*P* > 0.05), ** = *P* ≤ 0.01.

Preferences of both toad species depended significantly on vegetation structure (*B. bufo*: df = 2, χ^2^_2_ = 30.18, *P* < 0.001; *B. viridis*: df = 2, χ^2^ = 8.74, *P* = 0.01). Both species clearly avoided pools without macrophytes (*B. bufo*: df = 2, Wald = 16.33, *P* < 0.001, Fig. 1a; *B. viridis*: df = 2, Wald = 17.82, *P* < 0.001, Fig. 1b), and did not differentiate between either vegetation type (*B. bufo:* df = 2, Wald = 1.01, *P* = 0.32; *B. viridis*: df = 2, Wald = 2.06, *P* = 0.15). While *B. bufo* was not affected by the presence of fish (df = 1, χ^2^ = 0.061, *P* = 0.43), *B. viridis* significantly avoided fish pools (df = 1, χ^2^_1, 12_ = 9.79, *P* < 0.01).

The dragonfly *S. sanguineum* showed no preference for fish *vs*. fishless pools (df = 1, χ^2^_1, 12_ = 0.062, *P* = 0.80) or affinity for a specific vegetation type (df = 2, χ^2^_2, 8_ = 0.04, *P* = 0.98, Fig. 1c); whereas *S. danae* significantly avoided pools with fish (df = 1, χ^2^ = 7.01, *P* < 0.01, Fig. 1d), and preferred a specific vegetation type (df = 2, χ^2^_8_ = 8.12, *P* = 0.02). More precisely, fishless pools without macrophytes were significantly avoided (df = 1, Wald = 8.11, *P* < 0.01).

Both true bug species showed no significant preference for a specific vegetation type (*N. glauca*: df = 2, χ^2^ = 3.13, *P* = 0.209; *N. obliqua*: df = 2, χ^2^ = 3.28, *P* = 0.194). A significant interaction between fish presence and specific vegetation type was detected in both *N. glauca* and *N. obliqua* (df = 2, χ^2^ = 43.1, *P* < 0.001; df = 2, χ^2^ = 38.1, *P* < 0.001, respectively). Indeed, both species significantly preferred fishless pools with submerged and floating macrophytes (*N. glauca*: df = 2, Wald = 36.05, *P* < 0.001, Fig. 1e; df = 2, *N. obliqua*: Wald = 38.03, *P* < 0.001, Fig. 1f). Overall, however, only *N. glauca* was significantly less frequent in pools with fish (df = 1, χ^2^ = 6.20, *P* = 0.01), whereas *N. obliqua* was equally frequent in fish and fishless pools (df = 1, χ^2^ = 2.40, *P* = 0.12).

There was a significant interaction between fish presence and specific vegetation type in both diving beetle species *D. marginalis* and *A. sulcatus* (df = 2, χ^2^ = 16.10, *P* < 0.001; df = 2, χ^2^ = 26.23, *P* < 0.001, respectively). However, none of the species preferred any specific vegetation type (*D. marginalis*: df = 2, χ^2^ = 1.30, *P* = 0.523; *A. sulcatus*: df = 2, χ^2^ = 1.35, *P* = 0.510). As in the case of true bugs, both species significantly preferred fishless pools with submerged and floating macrophytes (*D. marginalis*: df = 2, Wald = 15.89, *P* < 0.001, Fig. 1g; *A. sulcatus*: df = 2, Wald = 25.52, *P* < 0.001, Fig. 1h), although neither completely avoided pools with fish (*D. marginalis*: df = 1, χ^2^ = 0.15, *P* = 0.70; *A. sulcatus*: df = 1, χ^2^ = 0.44, *P* = 0.51).

## Discussion

The present study reports similar responses to fish and vegetation structure within semiaquatic and aquatic organisms, rather than the expected preferences sorted primarily by specialization. In semiaquatic taxa (toads and dragonflies), habitat generalists did not distinguish between fish and fishless pools, whereas species specialized in fishless habitats selected fishless pools with their preferred vegetation structure, in accordance with our first hypothesis. However, all aquatic taxa (true bugs and beetles) significantly preferred fishless pools with submerged and floating macrophytes, regardless of the level of specialization. Therefore, each group relies on a different mechanism of predator detection and a different strategy for habitat selection.

In toads, only the specialist *B. viridis* significantly avoided fish-occupied pools, which corroborates the findings of previous studies on anurans specialized in fishless habitats^14,17,18^. Although this species naturally uses pools without fish and vegetation, in our study, it preferred fishless pools with macrophytes. Therefore, instead of vegetation structure, it likely uses a different mechanism for predator avoidance, such as chemical detection^29^. In adults, this mechanism must be reliable and strongly selected during evolution as the larvae, which inhabit ephemeral pools and rarely encounter fish, are typically palatable and unable to detect fish cues and react adequately to the danger of being devoured^67^. The preference of the generalist *B. bufo* for pools with macrophytes was unsurprising, as this species attaches its eggs to vegetation to prevent them from being washed away^42^. Although the preference of *B. viridis* for this type of pool was unexpected, the presence of vegetation offers some additional benefits, such as promoting the survival of the offspring by enabling for food growth (e.g., periphyton)^68^, as well as offering shade and refuge^69,70^.

A different strategy may be applied by the generalist *B. bufo*, who did not distinguish between fish and fishless pools. Habitats with fish typically have more periphyton and phytoplankton due to lower levels of herbivorous zooplankton and aquatic insects^5^. Pools with fish may also entail fewer competitors; therefore, as *B. bufo* larvae are toxic and unpalatable to fish^36^, it may be desirable to oviposit in fish-occupied pools. However, an exclusive preference for fish habitats could lead to overcrowding and negative density-dependent effects on offspring fitness^6,71^. As certain amphibians tend to avoid conspecifics, especially those with cannibalistic larvae^9,17,67^, *B. bufo* may favor an ideal free distribution to avoid a competitive environment for its larvae^72^. Indeed, species that can detect predators and conspecific density might adopt a mixed oviposition strategy and, like *B. bufo*, lay eggs in both predator-free and predator-occupied patches^73^.

Fish avoidance by a specialist was also observed in dragonflies, complementing evidence from natural experiments with Libellulidae^21,22^. In our study, only ovipositing *S. danae* significantly avoided fish-occupied pools. As chemical detection of predator cues has not been documented in adult odonates^74^, polarotaxis has been suggested as the main mechanism for habitat selection^27,75^, even though no evidence suggests it could have a role in predator detection. Alternatively, the presence of fish may alter water surface polarization patterns^27^, as regular feeding of fish causes eutrophication^76^, in turn, affecting habitat selection^21^. Although turbidity did not differ visibly between predator treatments, it is possible that differences were detected by *S. danae*.

The specialist *S. danae* showed a considerable preference for pools with macrophytes, which aligns with its natural preferences. In odonates, the attraction to a particular vegetation structure has been proposed as another possible mechanism for habitat selection^25^, which may serve as an indicator of predator presence. However, considering the mismatch we created between the fish presence and vegetation structure, this mechanism seems irrelevant. Some taxa susceptible to fish but unable to detect them, such as *Enallagma* spp. damselflies^20^ or the dragonfly specialist *Sympetrum depressiusculum*^77^, may rely on natal philopatry^78^. However, in our study, none of the animals emerged from the experimental pools. Fish detection and avoidance likely depend on more complex mechanisms, which will be determined by additional studies on other odonate species specialized in fishless habitats.

The generalist dragonfly *S. sanguineum* did not show predator avoidance or preference for a certain vegetation type. Based on evidence from the well-studied *Leucorrhinia* system, the larvae of dragonfly generalists may coexist with fish as they possess abdominal spines that provide defense against predation^38^ and may further elongate during ontogeny when fish are actually present in the environment^41^. They may also use behavioral defenses, such as burst swimming or a reduction in activity^46^. Despite the lower abundance of prey for odonate larvae in habitats with fish^5^, and consequent impact on fitness^79^, generalist dragonflies may resemble *B. bufo*, and spread their reproductive effort among fish-free and fish-occupied patches to avoid negative density-dependent effects on offspring. Such behavior has been described in mosquitoes^19,80^ in response to the actual presence of competitors, whereas in *S. sanguineum* the pools were completely free of competitors and there was only one ovipositing tandem pair at a time. Therefore, this could indicate risk-spreading^81^, whereby individuals unable to detect risk deposit their clutches among different habitat patches to increase the probability of offspring survival (i.e., bet-hedging). This was evidenced by tandem pairs of *S. sanguineum* ovipositing immediately after mating and spraying one clutch into several nearby pools. In contrast, oviposition of *S. danae* was preceded by flying around the net cage for a long time and careful selection of the suitable pool, in which the whole clutch was usually placed. Hence, spraying eggs among fish and fishless pools seems to be a general strategy to avoid predators and/or competitors only in species that can coexist with fish.

In both groups of aquatic insects, predator presence significantly affected habitat selection only in combination with vegetation structure: all species significantly preferred submerged and floating macrophytes in fishless pools but not in those with fish, regardless of their natural habitat preferences. In contrast, Binckley and Resetarits^1^ found no interaction between habitat complexity and fish presence during habitat selection by aquatic beetles. Moreover, the same diving beetles significantly selected fishless pools, regardless of their complexity. Food availability and quality, as well as plant community type (i.e., complexity) largely define dytiscid habitats^82^. Habitat complexity and prey density play important roles also in coexistence among true bugs. Vegetation provides shelter^83^ as well as a perch from which they capture their prey. Prey is less abundant in habitats with fish, which may hamper both backswimmers and beetles^5,13^. Surprisingly, only the generalist *N.* *glauca* was less frequent in pools containing fish. However, unlike the specialist *N. obliqua*, which can effectively exploit habitats with both low and high prey abundance, *N. glauca* needs high density of prey to achieve good feeding efficiency^84^.

Similar principles may have driven the habitat selection of diving beetles. Both study species are fast swimmers and seem to prefer open waters^85^. Therefore, fishless pools with submerged and floating macrophytes, which they preferred, may offer plenty of food and shelter, plus more space for movement than pools containing also littoral macrophytes. As with semiaquatic taxa, negative density-dependent effects (see above) or predator dilution effect^86^ may occur, whereby pools already containing other conspecifics may attract further colonists as adding prey reduces the overall predation risk. In contrast, large-bodied diving beetles such as *D. marginalis* employ secretions from their prothoracic and pygidial glands, which have narcotic and toxic effects on fish^37^, explaining the lack of selectivity for fish *vs*. fishless habitats.

In some taxa, both predator and dietary cues are needed to elicit full anti-predator responses^87,88^. In our study, the fish were caged; therefore, their chemical cues were present, but there was no signal of devoured conspecifics or heterospecifics. Thus, both backswimmer and beetle specialists would possibly avoid fish-occupied pools if fish posed a risk to them. This corroborates the finding that risk perception of certain aquatic taxa does not result from signals from predators alone, but from their consumption of prey^89^. As suggested by Åbjörnsson et al.^13^, this may not be true for the generalist *N. glauca*, which significantly avoided pools with fish cues.

In contrast, behavioral avoidance of some beetle taxa may be triggered by the mere presence of fish^1,5^ (but see^6^). Given that behavioral adjustments to the actual predator regime may be more important than complete avoidance of fish habitats^13^, it is possible that the consumptive effect of fish would elicit different behaviors in aquatic insects. Clear preference for pools with submerged and floating macrophytes without fish over those with fish points out to aquatic taxa relying mostly on chemical detection of the predator^13,19^. Habitat selection in aquatic taxa may be a complex process, as community assembly causes taxon-dependent feedback that alters fish avoidance behavior^6^. Tracking habitat selection behavior of marked individuals over time could help elucidate the underlying mechanism in these groups.

Our study expands current knowledge of habitat selection in response to a top predator by examining habitat selection behavior of taxa in relation to specialization and vegetation structure. Only specialists of semiaquatic taxa selected fishless habitats with vegetation structure matching their habitat preferences; whereas generalists relied on a bet-hedging strategy and/or responded to the actual presence of competitors. Therefore, oviposition habitat selectivity by semiaquatic specialists does not stem from their specialization to an inherent lack of fish, but from accurate predator recognition. In aquatic taxa, individuals probably respond to the actual risk of predation, regardless of specialization. Their preference for fishless pools with submerged and floating macrophytes probably stems from sufficient resources associated with this type of habitat. In specialists of semiaquatic groups, whose terrestrial adults use water only for breeding, the mere presence of a predator is sufficient to trigger avoidance; whereas in aquatic taxa, whose imagoes spend most of their lifetime in the water, the signal of consumed conspecifics/heterospecifics might be needed to elicit avoidance. Taxa with terrestrial adults and aquatic larvae obtain no feedback on the impact of adult decisions on the progeny; their mechanisms of habitat selectivity should therefore be faultless. The present results reinforce the importance of habitat selection for the colonization of aquatic ecosystems, and illustrate how taxa with different levels of specialization may respond differently to a top predator, depending on their life history. Future experiments using other generalists and a range of taxa from both sides of the specialist spectrum (e.g., species specialized in fishless as well as fish-heavy habitats) may help elucidate how widespread are the patterns found in this study, as well as which mechanisms animals use to avoid predators.

## Supplementary Information

Supplementary Information.

## Data Availability

The data that support the findings of this study are permanently archived in the figshare data repository under the link 10.6084/m9.figshare.16627561.v2.
