# Supplementary material for: Specialization directs habitat selection responses to a top predator in semiaquatic but not aquatic taxa
Source: Sci Rep. 2021 Sep 23;11:18928. doi: 10.1038/s41598-021-98632-2 (PMC8460784; doi:10.1038/s41598-021-98632-2)

Supporting Information

Figure S1. The design of the mesocosm experiment performed in four spatial blocks (A–D) of six pools each.

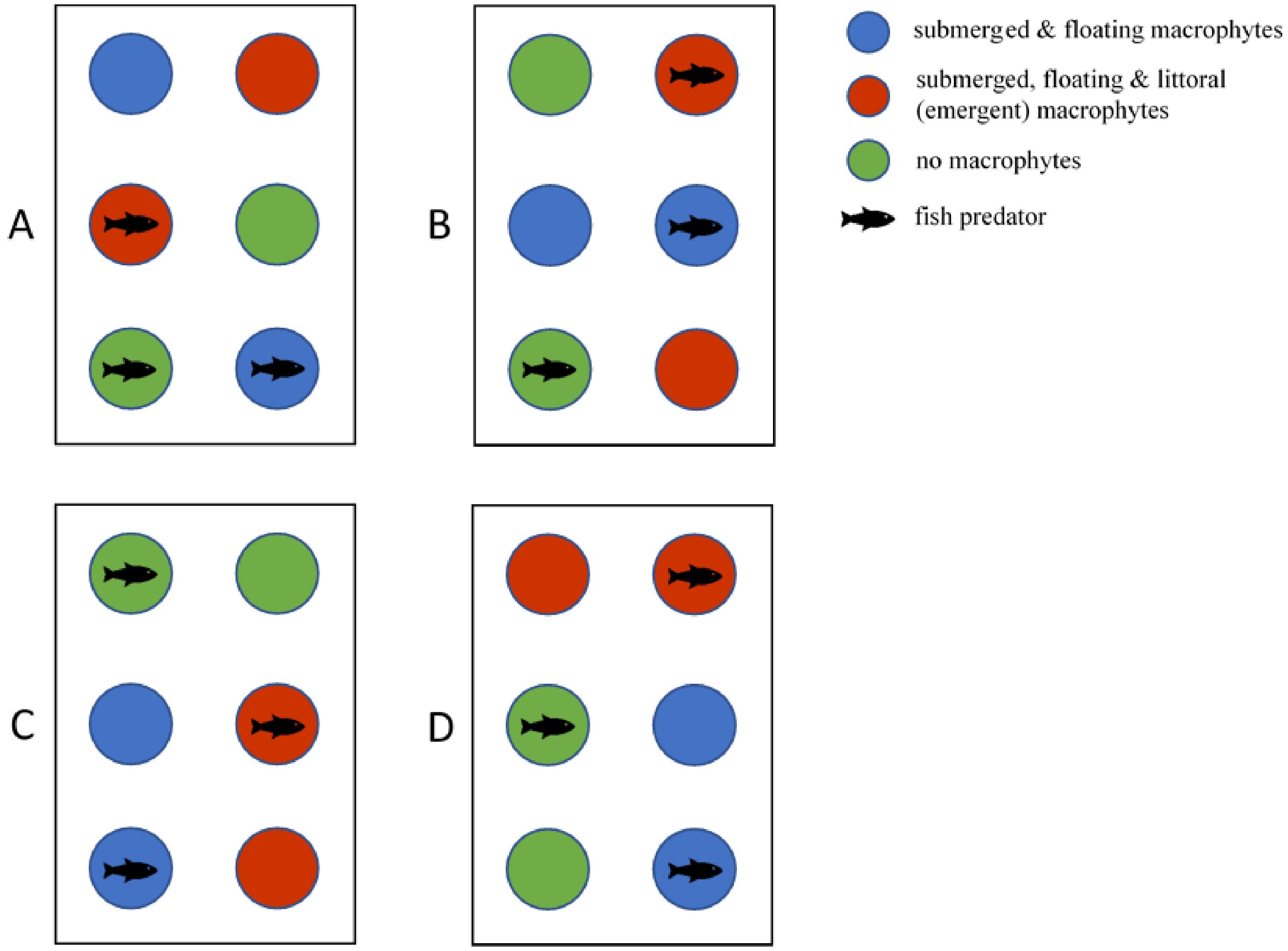

Figure S2. Detailed scheme of the block used for the mesocosm experiment. The pools are represented by circles.

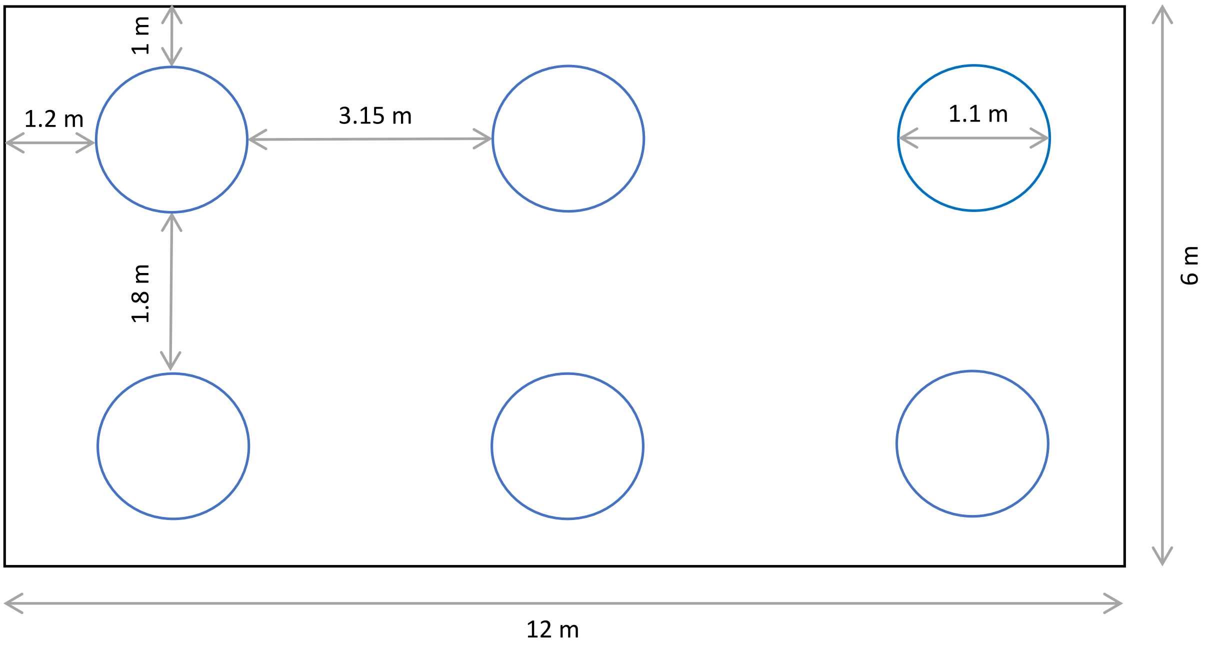

Supplement: Supplementary file 1 — Supplementary Information. [file 41598_2021_98632_MOESM1_ESM.pdf]
